# Nano-chitosan modified restorative materials suppress *Streptococcus mutans* biofilm and virulence gene expression

**DOI:** 10.1186/s13568-025-02004-2

**Published:** 2026-02-03

**Authors:** Jakline N. Saddik, Martha M. Naguib, Labib M. Labib, Ahmed O. El-Gendy, Fatma Molham

**Affiliations:** 1 Department of Microbiology and Immunology, Faculty of Pharmacy, Nahda University, Beni-Suef, Egypt; 2 Department of Microbiology and Immunology, Faculty of Pharmacy, Beni-Suef University, Beni-Suef, 62514 Egypt; 3 Department of Operative Dentistry, Faculty of Dentistry, Nahda University, Beni-Suef, Egypt

**Keywords:** Streptococcus mutans, Biofilm inhibition, Nano-chitosan (NCH), Antibacterial activity, Virulence genes, Oral microbiome

## Abstract

Secondary caries and biofilm production remain a persistent concern, prompting the development of antibacterial restorative materials. However, worries about synthetic additives’ safety and long-term effects urge the use of natural, biocompatible alternatives. This study explored the antibacterial efficacy of chitosan (CH) and nano-chitosan (NCH) incorporated into different restorative materials: amalgam (Am), glass ionomer cement (G), and resin composite (R). This study employs a direct comparative analysis of both modified and unmodified restorative materials against Am to evaluate their efficacy against *Streptococcus mutans* ATCC 25,175 using agar diffusion, broth inhibition, biofilm formation assays, and qPCR investigation of virulence genes (*ldh* and *gtfB*). Incorporating CH and NCH, especially at 15%, decreased biofilm biomass, downregulated virulence gene expression, and significantly increased the effectiveness of bacterial growth suppression. When compared to controls, the G modified with 15% NCH (GN15) showed the strongest inhibition of bacterial growth by 43.1%. The most significant finding was the ability of GN15 to downregulate the expression of *ldh* and *gtfB* by 50% and 28%, respectively, indicating a disruption in acid production and biofilm matrix synthesis. While Am showed the highest overall inhibition (86%, 65%). These results demonstrate that nano-chitosan is a biocompatible and promising addition to restorative materials, providing improved defense against cariogenic biofilms and facilitating the creation of dental biomaterials of the future.

**Supplementary Information:**

The online version contains supplementary material available at 10.1186/s13568-025-02004-2.

## Introduction

The oral cavity rich with around 700 bacterial species known as the oral microbiota (Larsen and Fiehn 2017; Rana et al., 2021). Its imbalance usually causes persistent dental problems (Molham et al., 2021). One of its principal members is *Streptococcus mutans*, it colonizes and adheres to the tooth surface forming biofilm. It can produce dental plaque, generate dental caries and periodontal diseases (Larsen and Fiehn 2017; Molham et al. 2021; Tahmourespour et al., 2011; Wen et al., 2010). *S. mutans* can break down carbohydrates forming lactic acid (Rana et al., 2021; Sangha et al., 2024; Tahmourespour et al., 2011), a key virulence feature controlled by the *lactate dehydrogenase* gene (*Idh*). Lactate production creates a hostile environment for competing oral bacteria, giving *S. mutans* a survival edge (Rana et al., 2021; Sangha et al., 2024; Tahmourespour et al., 2011). Furthermore, *S. mutans* secretes a variety of exoenzyme glycosyltransferases (GTFs), which are encoded by the *gtfB*, -*C*, and -*D* genes and are capable of cleaving the glucosyl moiety from sucrose to create glucans; these glucans act as a sticky matrix, it is the main building block in biofilm formation and the development of caries through *S. mutans* adhering firmly to tooth surfaces, forming dense plaque (Molham et al., 2021; Rana et al., 2021; Tahmourespour et al., 2011; Wen et al., 2010). Because bacterial biofilms are resistant to human defence systems and medications, their elimination are extremely difficult (Molham et al., 2021). A variety of interventions, such as phytochemicals, matrix-degrading enzymes, bacteriophages, and probiotics, were used to reduce or eliminate bacterial adhesion and biofilm development (Chhibber et al., 2013; Molham et al., 2021).

For more than 150 years, amalgam (Am), a restorative substance made of zinc (Zn), tin (Sn), silver (Ag), copper (Cu), and around 50% mercury (Hg), has been widely employed in dental applications because of its exceptional durability (WHO 2021; WHO 2009), significant antibacterial action (Beyth et al., 2007; Naguib et al., 2019; Savitri et al., 2016) with reduced bacterial adhesion (Kozmos et al., 2021). Researchers have demonstrated that bacteria developed resistance to mercury and antibiotics(Naguib et al., 2018, 2019; Summers et al., 1993; Wireman et al., 1997). Also, to safeguard human health and the environment from the discharge of hazardous compounds (Naguib et al., 2018, 2019; WHO 2021; WHO 2009), the World Health Organization recommended to phase-down Am usage and introduce mercury-free alternatives (WHO 2021; WHO 2009). The commonly used alternative restorative materials are Glass ionomer cement (G) and Resin composite (R). G is a biocompatible with a high capacity to release materials such as fluoride (Amin et al., 2021; Ibrahim et al., 2015; Labib et al., 2023; Weidlich et al., 2000). Although the low pH during setting and the release of fluoride ions were the reasons for the antibacterial properties of G (Amin et al., 2021; Ibrahim et al., 2015; Weidlich et al., 2000), G is still ineffective in influencing biofilm formation resulting in secondary caries (Hussein and Kareem 2024; Ibrahim et al., 2015; Shalaby et al., 2024). On the other hand, R application in dentistry has increased due to their better mechanical qualities (Ibrahim et al., 2015; Simonetta D’Ercole et al., 2022). Many investigations have indicated that the R material composition (Cazzaniga et al., 2017), surface roughness, charge (Simonetta D’Ercole et al., 2022; Song et al., 2015), and filler particle size and composition, all have a major impact on bacterial adherence (Ikeda et al., 2007; Motevasselian et al., 2017; Pereira et al., 2011; Simonetta D’Ercole et al., 2022). Accordingly, in addition to fluoride various additives have been incorporated into restorative materials (Amin et al., 2021, Hussein and Kareem 2024; Ibrahim et al., 2015; Labib et al., 2023; Lai et al., 2022; Petri et al., 2007; Tarsi et al., 1997a, b) such as silver nanoparticles (SN), quaternary ammonium compounds (QACs), chitosan (CH) and chitosan nanoparticles (NCH).

QACs provide a durable, non-leaching antibacterial surface, though their action is limited to direct contact (Antonucci et al., 2012; Imazato 2003). SN offers potent antibacterial activity, but they raise concerns for a potential cytotoxicity (Cheng et al., 2015; Corrêa et al., 2015). CH is a linear biopolyaminosaccharide produced from chitin, present naturally in crab and shrimp shells (Ikono et al., 2019; Muzzarelli 1977; Roberts 1992). It is no cytotoxic, biocompatible, antibacterial and antibiofilm (Ibrahim et al., 2015; Ikono et al., 2019; Tarsi et al., 1997a, b). On the other hand, NCH are expected to communicate with bacterial cells effectively because of their large surface area, charge density, and polycationic/polyanionic nature (Nasr et al., 2025). Previous reports have demonstrated antimicrobial activity of NCH, against microorganisms linked to dental caries, specifically *S. mutans* (Ikono et al., 2019; Labib et al., 2023; Lai et al., 2022) without cytotoxicity (Amin et al., 2021; Colonello et al., 2024; Fernandes et al., 2024; Ibrahim et al., 2015; Labib et al., 2023; Lai et al., 2022; Shalaby et al., 2024).

This study aimed to investigate different modified restorative material’s crucial role by assessing the effect of integrating traditional G and R with nontoxic CH and NCH on antibiofilm, antibacterial and growth efficacy of *S. mutans*. This study employs a direct comparative analysis of the efficacy of modified R and G against Am utilizing a high concentration of NCH (15%), furthermore this work provided deeper understanding of antibacterial and antibiofilm effect by evaluating molecular mechanisms to quantify virulence genes expression *ldh* and *gtfB.*

## Materials and methods

### Strains and reagents

#### Bacterial strains

The test microorganism used in this study was *Streptococcus mutans* ATCC 25,175 since it is directly involved in dental caries etiology (Larsen and Fiehn 2017; Tahmourespour et al., 2011; Wen et al., 2010). The stored strain at − 80 °C as glycerol stocks was initially recovered in Brain Heart Infusion (BHI) broth (HiMedia Laboratories, India) overnight at 37 °C for 24 h. under anaerobic conditions. Then, the broth culture was streaked onto BHI plates. For experimental use, a fresh single colony was inoculated into BHI broth and cultured at 37 °C for 48 h. in a candle jar.

#### Preparation of discs

G discs were prepared by combining the liquid and powder according to the manufacturer’s instructions. To get GN, the required proportion (w/w%) is made by integrating NCH powder (20 ± 5 nm) (NT-CSNP, NanoTech, Egypt for Photo-Electronics, Egypt) with G. On the other hand, G powder is mixed with CH powder (Sigma Aldrich, USA) to obtain the desired proportion (w/w%) of GC. The manufacturer instructed us to combine all the modified G powders with the G liquid. R discs were directly made from a ready-to-use syringe product. Additionally, to achieve the required proportion (w/w%) of the experimental RN and RC for the sample, NCH or CH and R are blended. Proportions are illustrated in Sup. 1 in supplementary file.

To shape the discs, the components were combined using a spatula on a glass slab in a semi-dark environment until a uniform consistency achieved. Discs (5 × 1 mm) were prepared by placing the mixture in 5 mm diameter standard Teflon molds enclosed between glass slides to attain a smooth surface. After solidification, all discs were sterilized using 95% ethanol and by exposure to ultraviolet (UV) light for 60 min. on each side (Li et al., 2010).

### Antibacterial susceptibility assay

#### Disc agar diffusion test (DAD)

The disc diffusion assay was used to measure the inhibition zones of the previously prepared and sterilized restorative materials Sup. 1 in supplementary file. The 200 µl of *S. mutans* bacterial suspensions made in sterile BHI broth were spread evenly with sterile cotton swabs throughout the BHI plate (Himedia, Mumbai, India). Tested discs (5 × 1 mm) were placed and fixed using sterile forceps in the BHI plate, including *S. mutans* bacteria. The plates were incubated at 37 °C for 48 h in candle jar. Visual inspection determined the diameter of the inhibition zone of the tested bacteria.

#### Liquid broth medium

*S. mutans* growth activity was measured in the presence of previously prepared and sterilized restorative materials Sup. 1 in supplementary file. *The S. mutans* bacterial suspension was prepared by transferring colonies into sterile BHI broth, with the turbidity adjusted to 0.5 McFarland. Next, 1 ml of the bacterial suspension was transferred into each well of the 24-well plate, which contained discs. The well without disc samples was the bacterial control, and the well without disc samples or M.O. was the media sterility control. Then all the treated bacterial samples were incubated in a candle jar at 37 °C for 48 h. All absorbance measurements (OD570) in this assay using Shimadzu UV-1201 UV/VIS spectrophotometer (Shimadzu, Kyoto, Japan). The bacterial growth inhibition rate was calculated using a previously published equation (Amin et al., 2021), as follows:

$$\:Inhibitory\:rate\left(\%\right)=\left(\frac{ODcontrol-ODmaterial}{ODcontrol}\right)X100$$

#### Bacterial growth curve

The antibacterial effect of the restorative materials was further evaluated by monitoring the growth kinetics of *S. mutans.* The restorative materials that have shown a high and significant *S. mutans’* growth inhibition were used for the assay. A bacterial suspension was prepared by transferring colonies into sterile BHI broth and adjusting the turbidity to 0.5 McFarland. Aliquots of 300 µl of bacterial suspension was transferred to every well of the 48-well plate, which contained discs. For comparison, wells without any of the discs and the well without any of the discs and M.O. were used as positive and negative control respectively. The plate was then incubated in a candle jar. These steps were done for 4 independent plates; each one was incubated for 12, 48, or 72 h. at 37 °C. Each disc was tested in triplicate (*n* = 3). After incubation, absorbance is measured at OD620 by Multiskan FC microplate readers ver. 1.01.16 (ThermoFisher, USA).

#### Biofilm activity assay

This assay was used to investigate material ability to interfere with biofilm formation, previously prepared and sterilized discs made of different restorative materials were used. Using a crystal violet (CV) staining assay, discs were placed into the wells of sterile, flat-bottom 96-well microtiter plates. *S. mutans* were initially grown on BHI agar. Then, it was incubated at 37 °C for 48 h. within a candle jar. A loopful colony was transferred into 5 ml of isotonic phosphate-buffered solution (PBS), till a turbidity adjusted to 0.5 McFarland’s dilution. The prepared isotonic bacterium mixture 10 µl was transferred to 10 ml of BHI broth. Then 200 µl of the isotonic bacterium-broth mixture transferred into a 96-well plate containing 100 µl of fresh broth + 0.1% sucrose and discs to be tested (wells without discs and wells with only sterile BHI broth were considered positive and negative control consequently). These steps were done for 4 independent microtiter plates; each one was incubated for 24, 48, 72, and 96 h. at 37 °C in a candle jar. Each disc was tested in triplicate (*n* = 3). At the end of incubation, the planktonic-containing media was removed from the plate, which was washed gently with PBS and stained using 0.1% CV dye (Adwic, Egypt) and then incubated at room temperature for 15 min. The stained cells attached to the wells were then washed with PBS and dried. Then, 200 µl of 95% ethanol (Adwic, Egypt) was added to each well to dissolve the crystal violet dye retained by the biofilm, and OD was measured at 620 nm.

### Virulence factor expression rate

#### Real-time qPCR

The relative quantitation of *gtfB* and *ldh* was made against 16s rRNA as a reference gene using the Stratagene MX3005P system (Agilent, USA) and QuantiTect SYBR Green PCR Master Mix Kit (Thermo Fisher, USA). The qPCR reaction mixture (25 µl) is mentioned in detail in Sup. 2 in supplementary file. All primers sequences used are summarized in Sup. 3 in supplementary file. PCR program conditions consisted of reverse transcription at 50 °C for 30 min., an initial denaturation at 94 °C for 15 min., then 40 cycles of amplification applied as follows: denaturation at 94 °C for 15 s., annealing at 60 °C for 30 s., and extension at 72 °C for 40 s., ending with one cycle of dissociation curve (1 cycle) as follows: denaturation at 94 °C for 1 min., annealing at 60 °C for 1 min., and final denaturation at 94 °C for 1 min. The critical threshold cycle (Ct) was defined by the Stratagene MX3005P as the cycle in which fluorescence becomes detectable above the background fluorescence and was inversely proportional to the logarithm of the initial number of template molecules.

For estimating gene expression variation of the different samples, the CT of each sample was calibrated relative to the control group (in the absence of restorative materials) according to the “ΔΔCt” method (Yuan et al., 2006) using the following ratio: (2-ΔΔct).

Whereas,

ΔΔCt = ΔCt reference gene − ΔCt target gene.

ΔCt target = Ct control − Ct material.

ΔCt reference = Ct control − Ct material.

The results were expressed as the means and standard errors of experiments.

### Statistical analysis

Statistical analysis was performed using R (version 4.4.3; R Core Team) (Team 2025) with different packages, including tidyverse (Hadley Wickham et al., 2019), car (Fox and Prince 2022; Weisberg 2019), emmeans (Lenth 2025), effectsize (Ben-Shachar et al., 2020), multcompView (Spencer Graves 2024), dplyr (Vaughan 2023), rstatix (Kassambara 2023b), ggplot2 (Wickham 2016), ggpubr (Kassambara 2023a), readxl (Bryan 2025), viridis (Garnier et al., 2024), patchwork (Pedersen 2024), tibble (Müller K 2023), and DescTools (Signorell 2025).

A one-way ANOVA test and post-hoc Dunnett’s test were used to understand the inhibitory effect. A two-way ANOVA test was used to understand the growth kinetics and biofilm formation of *S. mutans* and pairwise analysis using post-hoc Tukey and Bonferroni tests to compare effects between materials. For gene expression data, a one-way ANOVA followed by Tukey’s post-hoc test was conducted. Results were considered statistically significant at *p* < 0.05.

### RNA extraction

The gene expression assays were performed in the current study on previously prepared and sterilized G, GN15, and Am at time intervals that have shown significant biofilm formation of *S. mutans* three replicates. RNA extracted using the RNeasy Mini Kit (Qiagen, Germany, GmbH) according to manufacturer instructions. In summary, to protect RNA from degradation, a double volume of the RNA Protect Bacteria Reagent (Qiagen) was added to one volume of the tested broth culture; the mix was then vortexed and incubated for 5 min. at room temperature, then centrifuged for 10 min. at 8000 rpm. The supernatant was decanted. Then 200 µl of TE buffer (Thermo Fisher) containing lysozyme (1 mg/ml) (Biochemica, Applichem) was added to the pellet. Ethanol is then added to the lysate to promote selective binding of RNA to the RNeasy membrane. The sample was transferred to the RNeasy Mini spin column. Total RNA was bound to the column membrane, contaminants were efficiently washed away, and high-quality RNA was eluted in RNase-free water.

## Results

### Antibacterial susceptibility assay

#### Disc agar diffusion test (DAD)

All tested materials with *S. mutans* bacteria showed no inhibitory zones around the disc samples. A tiny halo measuring 1 ± 0.5 mm was observed exclusively surrounding the Am discs, consistent with findings from earlier research (Beyth et al., 2007; Hussein and Kareem 2024).

#### Liquid broth medium

**Fig. 1:**
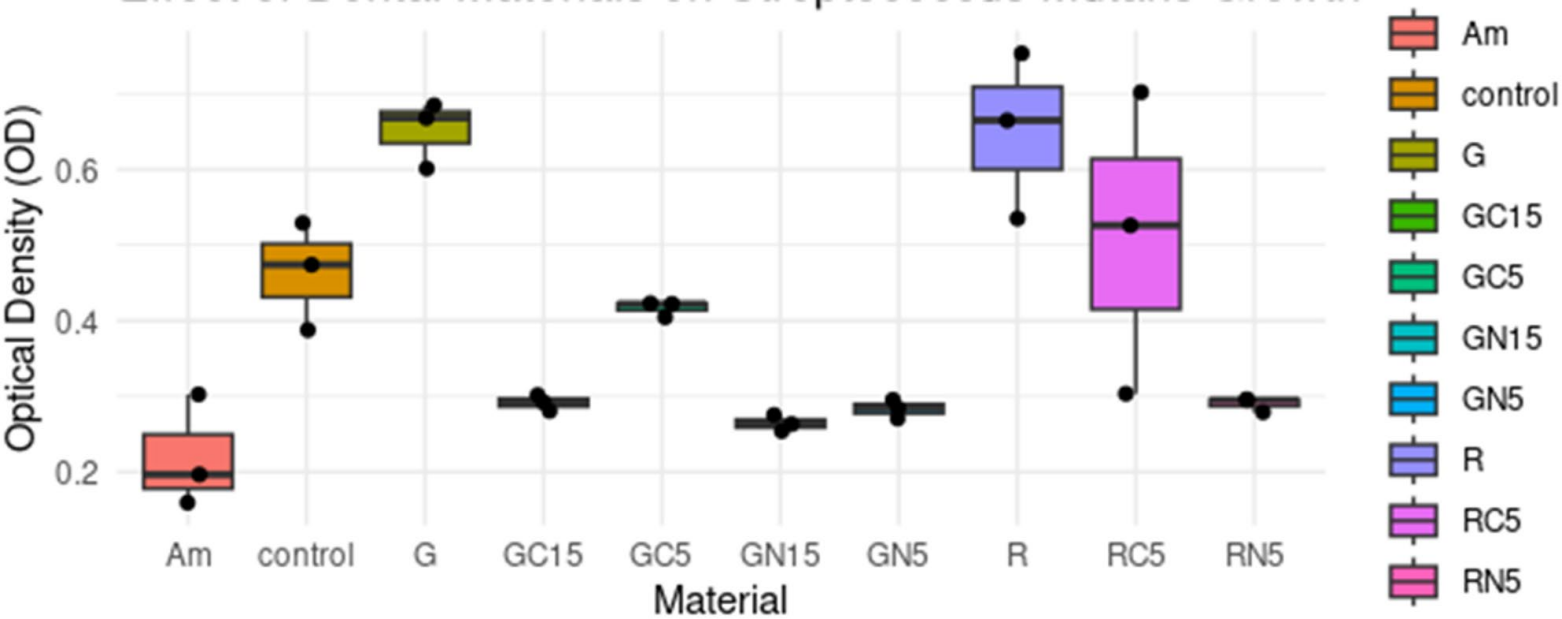
Illustrates the effect of different restorative materials: amalgam (Am), glass ionomer cement (G), NCH15% modified glass ionomer (GN15), NCH5% modified glass ionomer (GN5), CH15% modified glass ionomer (GC15), CH5% modified glass ionomer (GC5), resin composite (R), CH5% modified resin composite (RC5), and NCH5% modified resin composite (RN5)) on *S. mutans* growth

**Fig. 2:**
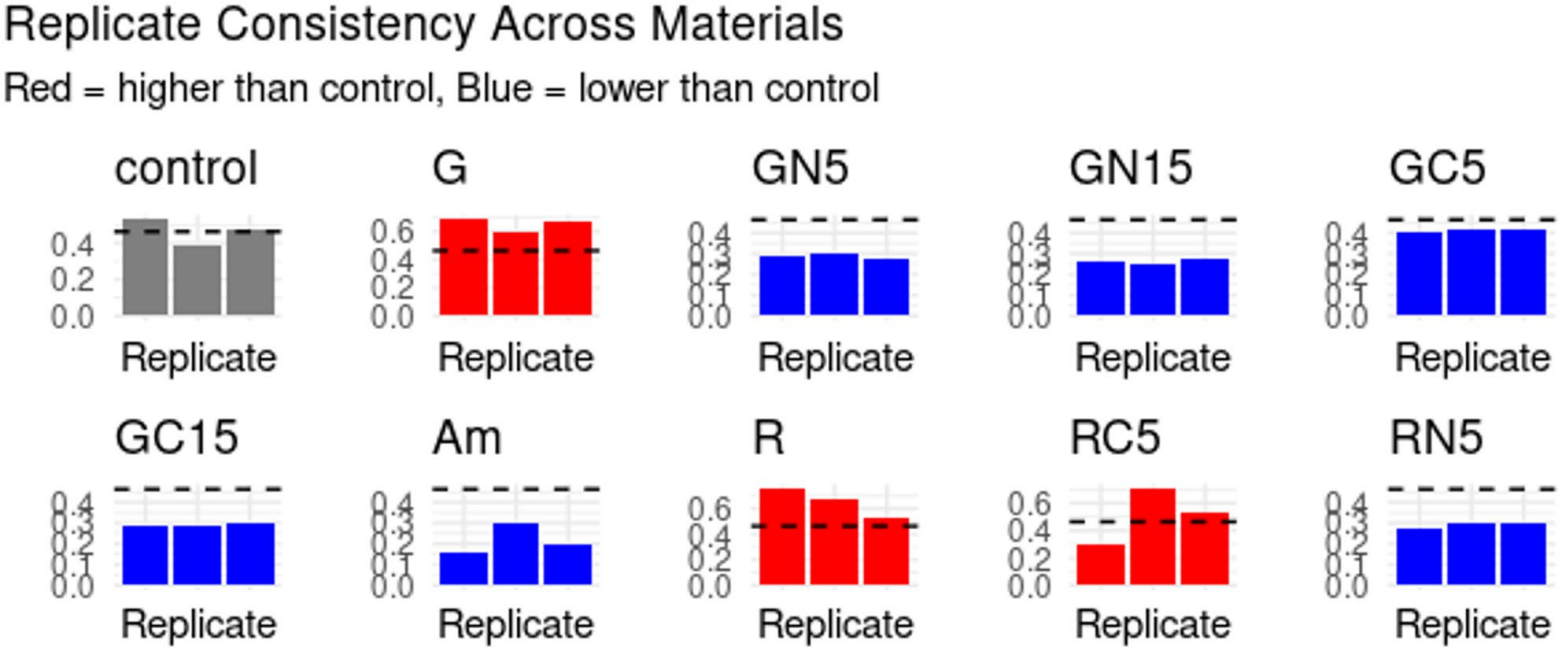
Illustrates the replicate consistency across each of the tested restorative materials: amalgam (Am), glass ionomer cement (G), NCH15% modified glass ionomer (GN15), NCH5% modified glass ionomer (GN5), CH15% modified glass ionomer (GC15), CH5% modified glass ionomer (GC5), resin composite (R), CH5% modified resin composite (RC5), and NCH5% modified resin composite (RN5). Gray refers to the control, red refers to restorative material with mean OD570 higher than control, and blue refers to restorative material with mean OD570 lower than control

Compared to Am, the most effective *S. mutans* inhibitor, G and R exhibited increased bacterial growth, which was effectively mitigated through modification with CH and NCH. NCH-modified restorative material demonstrated superior performance compared to CH-modified restorative material. Materials modified with NCH and CH concentrations exceeding 15% (20–30%) exhibited a significantly disrupted discs matrix, resulting in reduced surface strength; therefore, they were excluded from the experiments, as indicated in prior studies (Hussein and Kareem 2024; Ibrahim et al., 2015). The effects of GC5/GC15 exhibited greater variability compared to those of GN5/GN15. The NCH-modified restorative materials (GN5, GN15, RN5) consistently exhibited reduced bacterial growth compared to their base materials. The 15% NCH-modified restorative material (GN15) demonstrated greater efficacy compared to the 5% GN5. Analysis of the Table 1 indicates that materials exhibiting lower OD570 than the control wells which lacked restorative materials, including GN5, GN15, GC5, GC15, Am, and RN5, are likely to inhibit the growth of *S. mutans*. Both Am and GN15 demonstrated significant growth inhibition, at 52.58% and 43.1%, respectively. In contrast, restorative materials such as GN5, GC15, and RN5 exhibited strong, albeit not statistically significant, inhibition. The NCH-modified restorative materials (GN5, GN15, RN5) demonstrate consistently lower OD570 values. Notably, GN15 significantly inhibited growth (OD: 0.264 ± 0.011, -43.1%, *p* = 0.0424), indicating that NCH possesses antibacterial properties against *S. mutans*. In contrast to unmodified restorative materials such as G and R (OD570: 0.651 ± SD, + 40.3% vs. control;* p* ≃ 0.06) and RC5, which exhibited significant variability in results (CV = 39.1%), one replicate demonstrated markedly different behavior Table 1; Figs. 1 and 2.

**Table 1:** Summarize the mean OD570 for different restorative materials: amalgam (Am), glass ionomer cement (G), and NCH15% modified glass ionomer (GN15). Higher OD = more bacterial growth, SD = standard deviation (variability within replicates), SEM = standard error of the mean, and CV = coefficient of variation (relative variability)

Material	% inhibition rate	OD Mean ± SD	SEM	CV	*p*-value	ANOVA f-value	ANOVA *P*-value
Control	–	0.464 ± 0.071	0.041*1*	15.4	-	F (9,20) = 11.83	p = 0.000311*
G	+ 40.3%	0.651 ± 0.044	0.025*6*	6.81	0.0610		
GN5	− 38.7%	0.284 ± 0.012	0.007*14*	4.36	0.0766		
GN15	− 43.1%	0.264 ± 0.011	0.006*11*	4.00	0.0424*		
GC5	− 10.12%	0.417 ± 0.010	0.005*79*	2.41	0.9830		
GC15	− 37.03%	0.292 ± 0.011	0.006*05*	3.59	0.0977		
Am	− 52.58%	0.220 ± 0.074	0.042*9*	33.9	0.0094*		
R	+ 40.3%	0.651 ± 0.11	0.063*5*	16.9	0.0614		
RC5	+ 10.12%	0.511 ± 0.20	0.115	39.1	0.9835		
RN5	− 37.5%	0.290 ± 0.0097	0.005*58*	3.33	0.0934		

* indicates significant value

* p*-value, according to the post-hoc dunnett’s test

#### Bacterial growth curve

The assay was conducted on G, Am, and GN15, which had been previously prepared and sterilized. The two-way ANOVA test indicated a significant interaction effect between materials and time (F (9,32) = 54.41, *p* < 0.001, η² = 0.94), highlighting that the performance of materials on *S. mutans* growth differed across time points, as assessed by optical density (OD). Neither material (*p* = 0.924) nor time (*p* = 0.714) was significant independently. The interaction accounted for 94% of the variance in optical density (η² = 0.94, 95% CI [0.90, 1.00]).

**Fig. 3:**
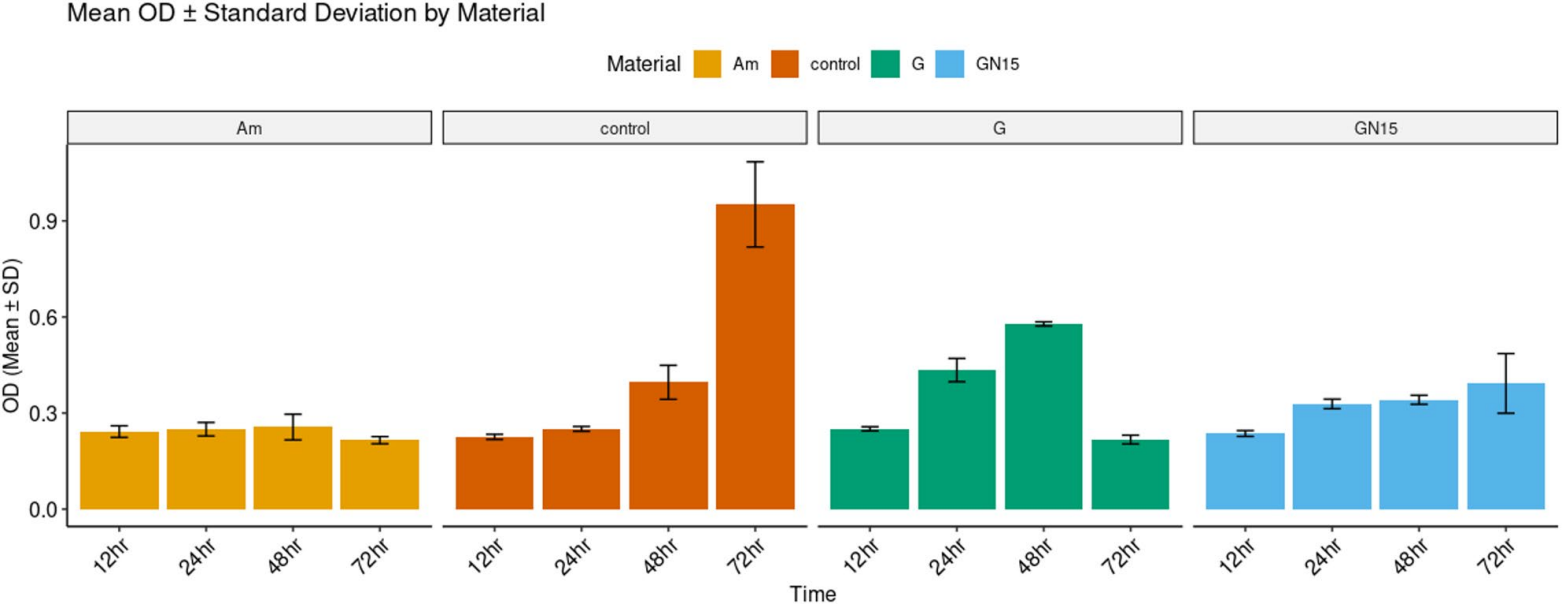
Bar plot of *S. mutans* growth at different time intervals (12, 24, 48, and 72 h.) in the presence of different restorative materials: amalgam (Am), glass ionomer cement (G), and NCH15% modified glass ionomer (GN15). Compare distributions (mean ± SD) for each material separately

**Fig. 4:**
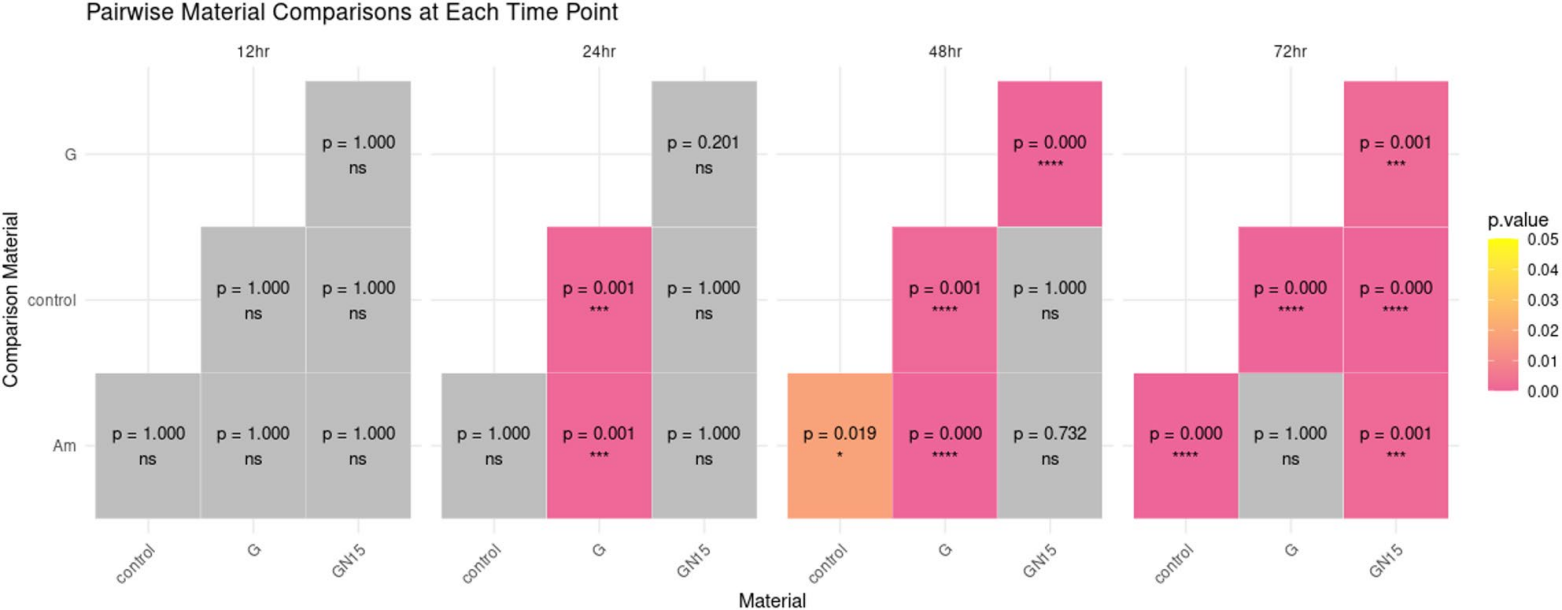
Pairwise comparisons of bacterial growth inhibition (optical density, OD) among different restorative materials: amalgam (Am), glass ionomer cement (G), and NCH15% modified glass ionomer (GN15) at 12, 24, 48, and 72 h. The heatmap shows p-values from post-hoc tests (Tukey). Darker colors indicate stronger statistical significance (****p < 0.0001; ***p < 0.001; **p < 0.01; *p < 0.05; NS = not significant). Lower OD values reflect better antibacterial performance. *N* = 3 replicates per group

Am and GN15 demonstrated a high inhibition rate, significantly reducing the growth of *S. mutans* by 52.58% and 43.1%, respectively. Am exhibited stable OD620 values (0.2–0.3) throughout all time points (*p* > 0.05 for time effect) and demonstrated lower bacterial growth (OD620), indicating better inhibition compared to G at most time points (24, 48 h.) (*p* < 0.05, Cohen’s d = 1.17) Figs. 3 and 4. GN15 demonstrated superior performance relative to G, exhibiting a notable decrease in OD620 at both 24 and 48 h. A significant enhancement was observed at 48 h. (*p* < 0.05, d = 0.38) in comparison to the unmodified G. G exhibited a decline in OD620 from 0.59 to 0.23 in the late stage, which was unexpected and indicates a delayed release of F^−^ beyond 48 h. Table 2; Figs. 3 and 4.

**Table 2:** Summarize the mean OD620 for the growth of *S. mutans* at different time intervals using different restorative materials: amalgam (Am), glass ionomer cement (G), and NCH15% modified glass ionomer (GN15) (higher = more bacterial growth), SD = standard deviation (variability within replicates), SE = standard error, median, IQR = interquartile range, Min = minimum, Max = maximum, confidence level = Cl-lower, Cl-higher, and Shapiro-Wilk* p*-value

Material	Time (hrs.)	*N*	Mean ± SD	SE	Median	IQR	95% CI for mean		Min	Max	Shapiro *P*-value
							Lower	Upper			
Control	12	3	0.225 ± 0.005	0.005	0.224	0.008	0.205	0.245	0.218	0.234	0.780
Control	24	3	0.250 ± 0.007	0.004	0.252	0.007	0.232	0.269	0.242	0.257	0.704
Control	48	3	0.396 ± 0.053	0.031	0.415	0.051	0.264	0.528	0.336	0.437	0.409
Control	72	3	0.951 ± 0.133	0.077	0.924	0.131	0.621	1.282	0.834	1.096	0.662
Am	12	3	0.242 ± 0.018	0.010	0.240	0.018	0.197	0.287	0.225	0.261	0.780
Am	24	3	0.250 ± 0. 021	0.012	0.240	0.019	0.197	0.302	0.235	0.274	0.236
Am	48	3	0.256 ± 0.040	0.023	0.240	0.0377	0.156	0.356	0.226	0.302	0.329
Am	72	3	0.215 ± 0.011	0.006	0.216	0.011	0.187	0.243	0.204	0.226	0.882
GN15	12	3	0.236 ± 0.005	0.005	0.239	0.009	0.214	0.259	0.226	0.244	0.533
GN15	24	3	0.328 ± 0.015	0.009	0.330	0.015	0.292	0.365	0.313	0.342	0.807
GN15	48	3	0.341 ± 0.008	0.008	0.338	0.014	0.306	0.377	0.329	0.357	0.631
GN15	72	3	0.393 ± 0.054	0.054	0.425	0.089	0.161	0.624	0.288	0.465	0.418
G	12	3	0.251 ± 0.006	0.004	0.249	0.006	0.235	0.267	0.245	0.258	0.603
G	24	3	0.434 ± 0.036	0.021	0.431	0.036	0.344	0.524	0.4	0.472	0.848
G	48	3	0.578 ± 0.004	0.004	0.578	0.007	0.562	0.595	0.572	0.585	0.967
G	72	3	0.218 ± 0.008	0.008	0.219	0.014	0.183	0.251	0.202	0.23	0.742

**Fig. 5:**
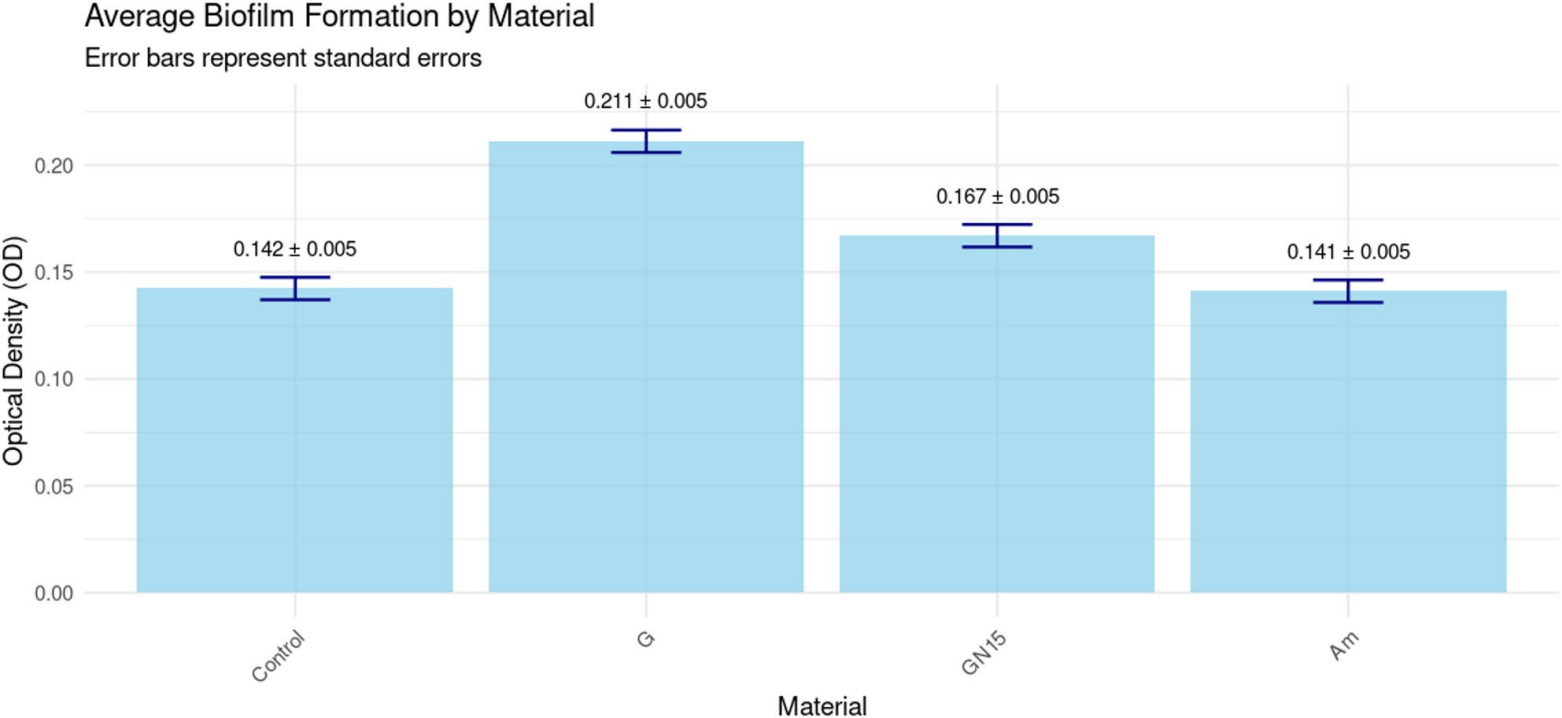
Average biofilm formation (optical density, OD) by different restorative materials: amalgam (Am), glass ionomer cement (G), and NCH15% modified glass ionomer (GN15). Points represent mean values (*n* = 12 measurements per material across all time points), and error bars show ± 95% confidence intervals. Letters indicate statistically homogeneous groups (Tukey’s HSD, *p* < 0.05)

The experiment was conducted on G, Am, and GN15. A two-way ANOVA demonstrated statistically significant main effects of material (F (3,32) = 4.561, *p* = 0.009, η² = 0.785), incubation duration (F (3,32) = 14.848, *p* < 0.001, η² = 0.709), and an interaction between material and time (F (9,32) = 2.594, *p* = 0.02, η² = 0.422). Material discrepancies are of paramount importance: 78.5% of the diversity in biofilm is attributable to material types. Compelling data indicates that at least one material diverges from the others and significantly influences biofilm formation (Am > GN15 > G in inhibition). Time has a strong significant effect (70.9%), so biofilm changes over time (peaking at 48 h), but the effect is smaller than material differences. Moderate interaction indicating that material performance was affected across time points on biofilm formation by *S. mutans*, as measured by OD620. Comparative analyses of biofilm formation among materials yield averaged results across time, with a confidence level of 95% as shown in Table 3. Employing the Tukey method to compare a set of four OD differences (estimated marginal means), Fig. 5 demonstrated significant disparities in biofilm production among the materials. G regularly exhibited markedly elevated OD in comparison to Am (*p* < 0.0001). The incorporation of 15% NCH into G (GN15) led to a quantifiable inhibition of biofilm (mean OD difference 0.044 ± 0.007, *p* < 0.0001), significantly decreasing biofilm formation, thus representing a valuable enhancement for patients at risk of caries. Am exhibited reduced biofilm accumulation compared to GN15 (mean OD difference 0.026 ± 0.007, *p* = 0.007).

**Table 3:** Descriptive statistics of biofilm optical density (OD) by material: amalgam (Am), glass ionomer cement (G), and NCH15% modified glass ionomer (GN15) and time

Material	Time (hrs.)	N	Mean ± SD	Median	SE	95% CI for mean		*P*-value	ANOVA* P*-value
						Lower	Upper		
Control	24	3	0.126 ± 0.004	0.126	0.002	0.115	0.136	0.961	*p* < 0.05
	48	3	0.203 ± 0.014	0.200	0.008	0.167	0.238	0.747	
	72	3	0.115 ± 0.011	0.114	0.006	0.088	0.141	0.88	
	96	3	0.126 ± 0.01	0.126	0.006	0.101	0.151	0.967	
G	24	3	0.173 ± 0.027	0.162	0.015	0.107	0.24	0.258	
	48	3	0.254 ± 0.026	0.242	0.015	0.191	0.318	0.152	
	72	3	0.208 ± 0.024	0.218	0.014	0.149	0.268	0.335	
	96	3	0.209 ± 0.012	0.216	0.007	0.178	0.24	0.046*	
GN15	24	3	0.131 ± 0.009	0.131	0.005	0.108	0.153	0.945	
	48	3	0.189 ± 0.019	0.189	0.011	0.142	0.237	0.983	
	72	3	0.167 ± 0.0126	0.170	0.007	0.135	0.197	0.436	
	96	3	0.182 ± 0.015	0.182	0.009	0.145	0.219	0.974	
Am	24	3	0.129 ± 0.020	0.137	0.012	0.079	0.179	0.369	
	48	3	0.162 ± 0.023	0.173	0.013	0.106	0.219	0.194	
	72	3	0.125 ± 0.020	0.127	0.012	0.075	0.175	0.840	
	96	3	0.148 ± 0.024	0.146	0.014	0.088	0.208	0.866	

* significant value

Values represent mean ± standard deviation (SD) with median [interquartile range, IQR]. Normality was assessed via the Shapiro-Wilk test (*p* < 0.05)

**Fig. 6:**
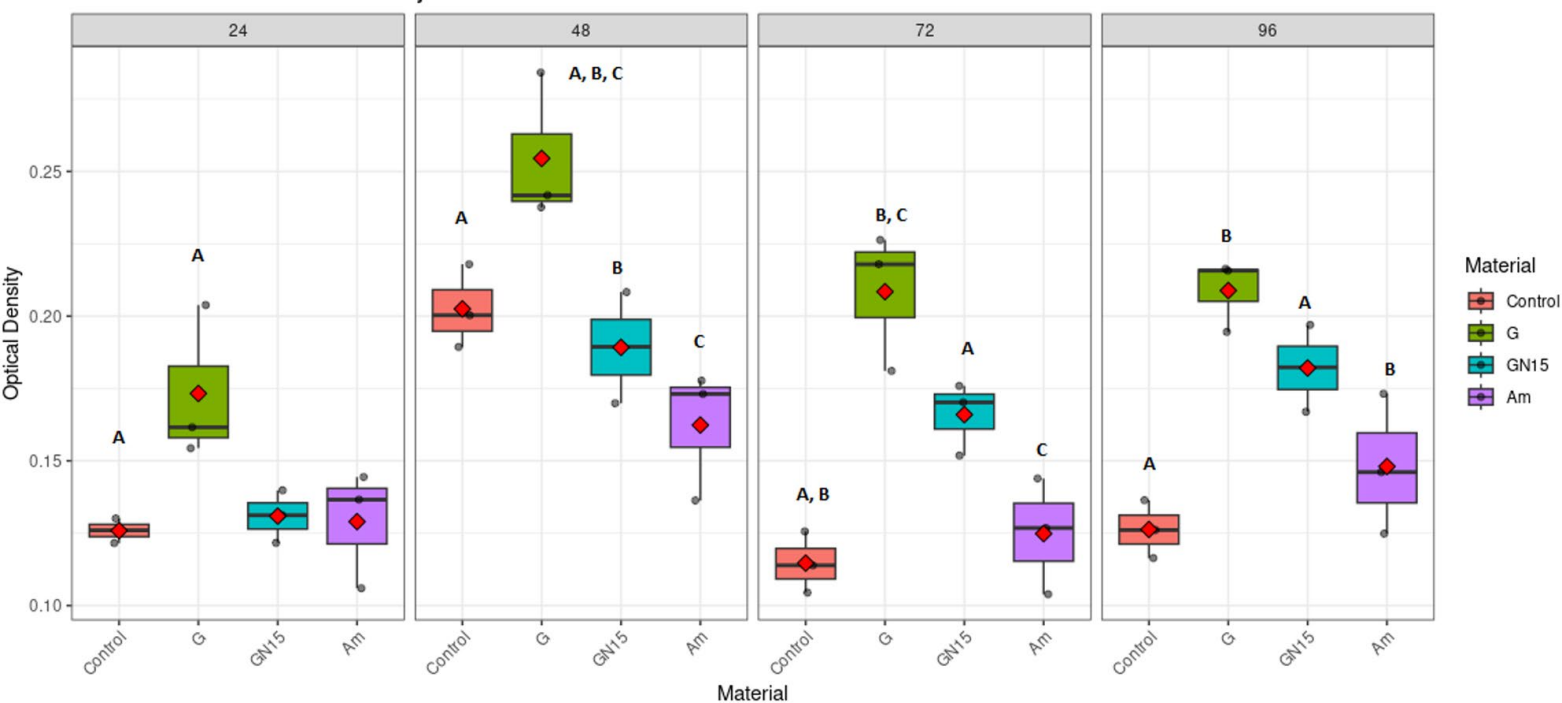
Distribution of *S. mutans* biofilm formation (optical density, OD) by restorative materials: amalgam (Am), glass ionomer cement (G), and NCH15% modified glass ionomer (GN15) and incubation time. Boxplots show median (center line), interquartile range (box limits), and full data range (whiskers) with individual data points overlaid (*n* = 3 replicates per group). Letters indicate significant differences (Tukey’s HSD, *p* < 0.05) between materials within each time point

Comparisons at each time point revealed dynamic patterns of biofilm formation Fig. 6. At 24 h., G already showed higher biofilm accumulation than GN15 and Am but did not differ significantly (all pairwise *p* > 0.05). By 48 h., material differences became more pronounced, with G (0.254 ± 0.026) > GN (0.189 ± 0.019) > Am (0.162 ± 0.023) in biofilm accumulation, showing a significant difference in biofilm formation between (G and GN15) and (G and Am) (*p* < 0.05). This pattern persisted at 72 and 96 h., G > GN > Am; biofilm levels declined across all materials, with G again showing significantly higher accumulation than Am (*p* ≤ 0.006). The GN15 showed intermediate values that did not differ significantly from either G or Am (*p* > 0.05).

#### Virulence factor gene expression rate

**Fig. 7:**
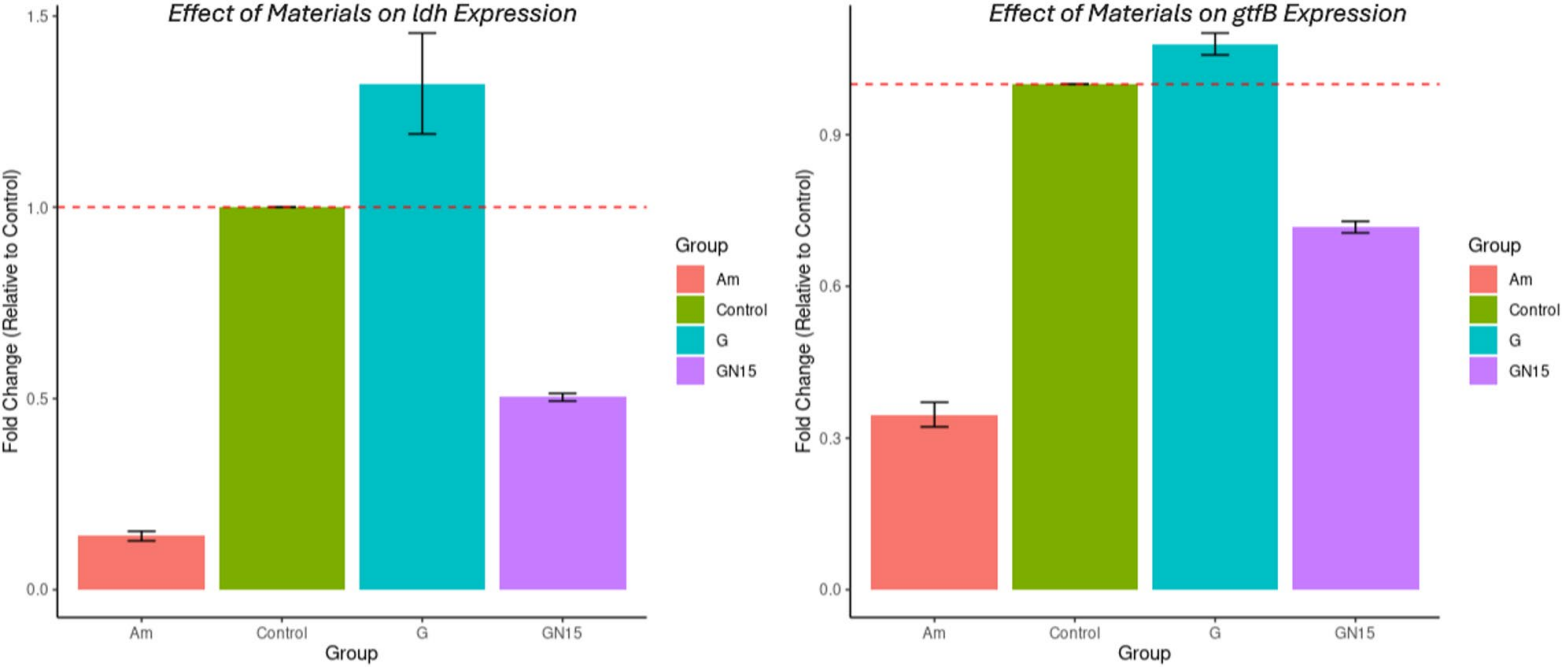
Boxplots showing *ldh* and *gtfB* expression of *S. mutans* in the presence of different dental restorative materials: amalgam (Am), glass ionomer cement (G), and NCH15% modified glass ionomer (GN15). The red dashed line indicates control levels

Quantitative Real-time RT-PCR was used to quantify the effect of different dental restorative materials on the *gtfB* and *ldh* gene expression of the *S. mutans* ATCC25175 biofilm, using the 16 S rRNA gene as a reference. RT-PCR revealed significant different expression of virulence genes (*ldh* and *gtfB*) in *S. mutans* following treatment with experimental materials (Am, GN15, G) compared to the untreated control Fig. 7, Sup. 4 in supplementary file. The 86% suppression of *ldh* by Am (0.14 ± 0.02; *p* < 0.001) suggests near-complete disruption of lactic acid fermentation, while G’s slight 32% upregulation (1.32 ± 0.23; *p* = 0.035) of biofilm possibly reflects stress. On the other hand, adding 15% NCH to G (GN15) changed the effect from 31% upregulation to 50% downregulation of *ldh* gene expression (0.50 ± 0.02; *p* = 0.003) and may confer therapeutic benefit in reducing biofilm formation. For the effect of the materials on the *gtfB* gene level, Am’s 65% reduction (0.35 ± 0.04; *p* < 0.001) indicates a profound disruption of bacterial adhesion. The null effect of G (1.08 ± 0.04; *p* = 0.045) suggests structural modifications (as in GN15) are required for anti-biofilm activity; it showed significant 28% gene suppression (0.72 ± 0.02; *p* < 0.001).

## Discussion

The present study evaluated the efficacy of different CH- or NCH-modified or unmodified restorative materials (Am, R, G) against *S. mutans* (ATCC 25175). These bacteria are closely linked to biofilm formation and the development of oral diseases (Victor Alves Carneiro et al., 2019).​.

The disc agar diffusion test (DAD) showed false negatives with inhibition halos smaller than 2 mm, which aligns with previous findings that conventional glass ionomer cement often shows no activity in this test (Beyth et al., 2007; Hussein and Kareem 2024). This may be because the agar diffusion test was mainly designed for antibiotics and is influenced by factors like the material diffusion rate in agar and interactions between the material and agar (Nunes et al., 2020). In contrast, our results show that Am performs better in broth by significantly reducing bacterial growth (measured as OD values) compared to all other modified and unmodified materials (R, RN, RC, G, GN5, GN15, GC5, and GC15) when added to broth media for 48 h. Modification with CH and NCH successfully improved both the unmodified glass ionomer G and resin composite R, demonstrating superior bacterial growth. NCH-modified restorative material generally outperformed CH-modified restorative material. The 15% NCH- and CH-modified restorative material was more effective than the 5% NCH- and CH-modified restorative material.

Both Am and GN15 significantly inhibited the growth (-52.58% and − 43.1%, respectively), while restorative materials such as GN5, GC15, and RN5 showed strong (but not significant) inhibition. The NCH-modified restorative materials (GN5, GN15, and RN5) consistently showed significant growth, suggesting NCH has antibacterial properties against *S. mutans*. Unlike unmodified restorative materials such as G and R. The greater bacterial growth, adhesion, and biofilm deposition on unmodified restorative materials vs. CH- and NCH-modified G; although it defies logic, the study’s observation of *S. mutans* growing more by G is biologically believable. The key factors and processes that may account for this interaction are nutrient gradients, redox potential, rough and porous surfaces, additive content, and pH (Arora et al., 2022). Other studies indicate that time and additives added to G have an impact on the fluoride ions (F^−^) leaching. Compared to modified G with CH or NCH, the F^−^ release from G was much lower; nonetheless, the release increased with time (Kirthika et al., 2021; Labib et al., 2023; Nicholson et al., 2023). Additionally, the surface roughness increases the surface area available for bacterial protective micro-niches and adherence (Montanaro et al., 2004; Poggio et al., 2018). G’s elevated surface roughness coupled with the surface integrity loss brought on by fluoride leakage makes it more vulnerable to bacterial growth and adhesion development (Arora et al., 2022; Kirthika et al., 2021). Additionally, regarding pH dynamics, fresh G has an acidic pH during its setting phase, while *S. mutans* thrives at a pH range of 4.5 to 7.0 (Baker et al., 2017; Saputra et al., 2019). Within 24–48 h., the temporary pH of G rises to neutral, creating optimal growth conditions, which may reduce its antibacterial efficacy (Saputra et al., 2019).

The antibacterial properties of CH- and NCH-modified dental fillings include strategies to reduce growth on unmodified dental fillings, such as cationic bacterial membrane disruption occurring by the amino (NH₃⁺) groups of CH; in addition, CH decreases surface porosity (reduces roughness) and enhances surface flexure strength, and CH forms chelates with essential metal ions required for the metabolic enzymes of bacteria (Kirthika et al., 2021; Labib et al., 2023). Furthermore, NCH-modified dental fillings (e.g., GN5 and GN15) outperformed CH-modified dental restorative materials (e.g., GC5 and GC15), which can be explained by their greater surface-to-charge density, larger surface area, and enhanced cellular uptake (Poznanski et al., 2023).

The current study revealed, considering the antibacterial activity over time, that Am exhibited superior efficiency with persistent antibacterial activity, outperforming G and GN15 at 24, 48, and 72 h. This evidence aligns with earlier studies demonstrating that Am’s long-term ion release (Hg, Cu^2+^, Ag^+^, etc.) hinders bacterial adhesion (Gul et al., 2023; Lussi et al., 1990; Orstavik 1985). Am’s OD620 demonstrated consistency from 0.20 to 0.3 across all measured time points (*p* > 0.05 for time impact), indicating that it can be relied upon for long-term restorations in patients with a high risk of caries. The interaction between material effect on growth and time accounted for 94% of the variance in optical density (η² = 0.94, 95% CI [0.90, 1.00]), suggesting significant variability in material performance across time points.

Furthermore, GN15 exhibited enhanced performance compared to unmodified G, exhibiting a notable OD620 reduction at 24 and 48 h., with significant improvements (*p* < 0.05) recorded at the 48 h. time interval in comparison to unmodified G. Additionally, it indicated that the stable OD620 ranged from 0.31 to 0.465 at 24, 48, and 72 h. Conversely, G, without modifications, exhibited unusual behavior, with over 60% of OD620 diminishing from 0.59 to 0.23 at time intervals of 48 to 72 h. The postponed release of F⁻ may account for the enhanced observed antibacterial efficacy in the later stages. Many earlier investigations have indicated that the release of F⁻ from the unmodified G is a complex process, requiring a variable duration (from 48 h. to 7 days) to effectively demonstrate its antibacterial activity (Labib et al., 2023; Montanaro et al., 2004; Poggio et al., 2018) and is influenced by several factors previously mentioned, including the cement formulation (De Witte et al., 2000; el Mallakh 1990; Hörsted-Bindslev and Larsen 1990), the physiochemical properties of the cement’s setting reaction, and the pH of the medium (De Witte et al., 2000; el Mallakh 1990; Forsten 1990; Morales-Valenzuela et al., 2020).

The effectiveness of modification to inhibit biofilm formation was evaluated in the current study by incorporating 15% NCH into G. This modification resulted in a clinically meaningful reduction in the formation of *S. mutans* biofilm, achieving statistical significance (*p* < 0.001). The modification significantly inhibits the formation of biofilms, as indicated by the observed effect size (d = 1.4). This information adds credence to the increasing amount of evidence showing that G modifications can improve the antimicrobial qualities of dental restorative materials by interacting with bacterial cell membranes through NCH positive charge interaction (Hussein and Kareem 2024), entering bacterial cells to bind bacterial DNA, and thereby disrupting the central dogma (Hussein and Kareem 2024; Labib et al., 2023).

A prior study found that a gradual increase in NCH solution in the G liquid at v/v ratios enhanced the antibacterial efficacy of G against *S. mutans* without compromising bacterial adhesion; however, the incomplete biofilm suppression when compared to Am indicated that a 15% concentration might not be enough for total prevention (Yilmaz Atay 2020). While Am demonstrated numerically significantly lower biofilm accumulation compared to GN15 (*p* = 0.007), the medium effect size (d = 1.02) suggests the difference is clinically meaningful. This performance from Am’s well-documented continuous mercury ion release (Okabe et al., 2003), which provides sustained antimicrobial-antibiofilm activity (Hegde et al., 2018). Am continues to be the industry leader in biofilm prevention, consistently demonstrating the lowest biofilm formation. Am is preferred when permissible; the GN15’s relatively comparable performance is notable given increasing mercury avoidance trends (WHO 2021), suggesting NCH modification could develop into a viable alternative where Am use is contraindicated.

RT-PCR revealed significant differential expression of virulence genes (*ldh* and *gtfB*) in *S. mutans* following treatment with experimental materials (Am, GN15, G) compared to untreated controls Fig. 7, Sup. 4 in supplementary file. Targeting the *ldh* gene is clinically relevant, as it is a major virulence factor in *S. mutans* (Rana et al., 2021; Sangha et al., 2024; Tahmourespour et al., 2011), linking sugar metabolism to acid production (caries development) and biofilm competitiveness (plaque dominance) (Sangha et al., 2024; Ye et al., 2025). Our data shows Am strongly suppresses *ldh* (86% downregulated, *p* < 0.001), making it the strongest candidate for anti-caries strategies. In contrast, G slightly upregulated *ldh* (*p* = 0.035), but when G was modified with NCH (GN15), *ldh* was downregulated by 50% (*p* = 0.035). Inhibiting *ldh*, as seen with Am and GN15 in our study, could reduce acid production, preventing cavities; weaken *S. mutans* survival, as acidic conditions can disrupt biofilm formation; and lower bacterial competitiveness, promoting a healthier oral microbiota.

Targeting *gtfB* is clinically significant as a master regulator of *S. mutans* biofilm formation (Molham et al., 2021), making it a prime target for anti-caries therapies and plaque-control strategies. Without *gtfB*, the bacterium loses its ability to adhere and colonize teeth efficiently (Tahmourespour et al., 2011; Wen et al., 2010); cannot form aggregations with other bacteria (Conrads et al., 2014; Zhu et al., 2023); and has less protection from saliva flow, antimicrobial agents, and immune defences (Kawada-Matsuo and Komatsuzawa 2017; Lemos et al., 2019). Inhibiting *gtfB*, as seen with Am & GN15 in our study, can reduce biofilm formation, suggesting the importance of structural modifications (as in GN15) for anti-biofilm activity. Our data showed Am had the strongest *gtfB* suppression (65% reduction, *p* < 0.001) and the best anti-biofilm effect. G had no significant impact on *gtfB* (which remained near baseline, *p* = 0.045); however, adding NCH in GN15 improved this, resulting in a 28% reduction effect (*p* < 0.001).

The present study indicates that Am remains the most dependable choice for long-term caries prevention in high-risk scenarios, owing to its proven clinical durability and significant reduction of biofilm formation. Its status as a benchmark is earned through over a century of documented clinical success and exceptional wear resistance. However, its elevated mercury content drives the imperative for effective alternatives. The NCH modification significantly enhances the antibiofilm performance of G, and while GN15 has not yet reached the comprehensive efficacy of Am, it offers a promising mercury-free alternative. For patients at risk of caries, modified GICs like GN15 present a superior option to unmodified G, particularly in pediatric or aesthetically sensitive cases where Am is contraindicated or not preferred. Ultimately, material selection must integrate antibacterial properties with aesthetic considerations, biocompatibility, and long-term mechanical performance.

### Limitations

There are certain limitations to this in vitro investigation. In vitro conditions may not fully replicate oral environments. The oral environment is continuously under dynamic stress due to the presence of other oral microbes and the consumption of different foods and drinks. So, more investigation is required using a system similar to the salivary content and oral cavity circumstances of dental restorations. More evaluations is needed to assess the mechanical properties post-modification, particularly for CH- and NCH-modified restorative materials with concentrations higher than 5%.

### Prospects

Evaluation of 15% nanochitosan-modified glass ionomer cement effects on other cariogenic species using longitudinal clinical trials.

## Supplementary Information

Below is the link to the electronic supplementary material.

Supplementary Material 1.

## Data Availability

All data generated or analyzed during this study are included within this published article.
