# Supplementary material for: Nano-chitosan modified restorative materials suppress Streptococcus mutans biofilm and virulence gene expression
Source: AMB Express. 2026 Feb 3;16:20. doi: 10.1186/s13568-025-02004-2 (PMC12920852; doi:10.1186/s13568-025-02004-2)
Supplement: Supplementary file 1 — Supplementary Material 1. [file 13568_2025_2004_MOESM1_ESM.docx]

**Nano-Chitosan Modified Restorative Materials Suppress *Streptococcus mutans* Biofilm and Virulence Gene Expression**

**Jakline N. Saddik^1†^, Martha M. Naguib^2†^, Labib M. Labib^3^, Ahmed O. El-Gendy^2^, Fatma Molham^2^***

**^1^ Department of Microbiology and Immunology, Faculty of Pharmacy, Nahda University, Beni-Suef, Egypt.**

**^2^ Department of Microbiology and Immunology, Faculty of Pharmacy, Beni-Suef University, Beni-Suef 62514, Egypt.**

**^3^ Department of Operative Dentistry, Faculty of Dentistry, Nahda University, Beni-Suef, Egypt.**

**†** These authors contributed equally to this work.

***Correspondence**

Fatma Molham

Microbiology and Immunology Department, Faculty of Pharmacy, Beni-Suef University, Egypt

E-mail: [Fatma.molham@pharm.bsu.edu.eg](mailto:Fatma.molham@pharm.bsu.edu.eg)

Postal address: Beni-Suef, 62514

**Sup. 1** Summarize the restorative materials, additives used to modify the restorative materials, ratio between restorative materials and the added modifier used throughout the study.

| Brand name/ company | Material: modifier ratio (mg: mg) | Abbreviation | Material used (w/w%) |
| --- | --- | --- | --- |
| Nexcomp set, Meta Biomed Euro GmbH, Germany | 1000 ® | R | Unmodified Resin composite cement |
|  | 950:50 (R: NCH) | RN5% | Resin composite modified with 5% nanochitosan |
|  | 950:50 (R: CH) | RC5% | Resin composite modified with 5% chitosan |
| Medifil code no 2603, Promedica Dental Material GmbH, Germany | 1000 (G) | G | Unmodified glass ionomer cement |
|  | 950:50 (G: CH) | GC5% | Glass ionomer cement modified with 5% chitosan |
|  | 850:150 (G: CH) | GC15% | Glass ionomer cement modified with 15% chitosan |
|  | 700:300 (G:CH) | GC30% | Glass ionomer cement modified with 30% chitosan |
|  | 950:50 (G: CH) | GN5% | Glass ionomer cement modified with 5% nanochitosan |
|  | 850:150 (G: CH) | GN15% | Glass ionomer cement modified with 15% nanochitosan |
|  | 700:300 (G: CH) | GN30% | Glass ionomer cement modified with 30% nanochitosan |
| GS-80 alloy amalgam caps, SDI, Australia | (alloy /amalgam)  (Ag, Sn, Cu/ Hg)  (1/0.9)  (800/720mg)  Alloy (Ag 40%, Sn31.3%, Cu28.7%)  Prepared according to manufacturer’s instructions | Am | Amalgam |

**Sup. 2** Summarize qPCR reaction components used for *16S rRNA*, *gtfB*, and *ldh* genes expression

| Component | Volume/reaction | Producer |
| --- | --- | --- |
| 2x QuantiTect SYBR Green PCR Master Mix | 12.5μl | Thermo Fisher, USA |
| Reverse transcriptase | 0.25μl | Thermo Fisher,USA |
| Forward primer (20 pmol) | 0.5μl | Willowfort, UK |
| Reverse primer (20 pmol) | 0.5μl | Willowfort, UK |
| RNase Free Water | 8.25μl | Thermo Fisher, USA |
| Template RNA | 3μl |  |
| Total | 25μl |  |

**Sup. 3** Summarize oligonucleotides sequences used as primers for *16S rRNA*, *gtfB*, and *ldh* genes expression

| Target gene | Primer sequence (5'-3') | Reference |
| --- | --- | --- |
| *Ldh* | TTGGCGACGCTCTTGATCTTAG  GTCAGCATCCGCACAGTCTTC | (Wen et al. 2010) |
| *gtfB* | AGCAATGCAGCCAATCTACAAAT  ACGAACTTTGCCGTTATTGTCA | (Tahmourespour et al. 2011) |
| 16S rRNA | CCTACGGGAGGCAGCAGTAG  CAACAGAGCTTTACGATCCGAAA |  |

**Sup. 4** Gene expression fold changes (mean ± SD) and statistical significance (post-hoc tests vs. control) in the presence of dental restorative materials: Amalgam (Am), glass ionomer cement (G), and NCH15% modified glass ionomer (GN15)

| Group | Gene | n | Mean± SD | Post-hoc  P-value | ANOVA f-value | ANOVA p-values | Interpretation |
| --- | --- | --- | --- | --- | --- | --- | --- |
| Am | *gtfB* | 3 | 0.35±0.04 | <0.001 | *gftB*  370.9  *ldh*  62.17 | <0.001 | Best gene suppression |
|  | *ldh* | 3 | 0.14±0.02 | <0.001 |  |  |  |
| G | *gtfB* | 3 | 1.08±0.04 | 0.045 |  |  | Moderate gene suppressor |
|  | *ldh* | 3 | 1.32±0.23 | 0.035 |  |  |  |
| GN15 | *gtfB* | 3 | 0.72±0.02 | <0.001 |  |  | Highest gene expression |
|  | *ldh* | 3 | 0.50±0.02 | 0.003 |  |  |  |

**References**

Tahmourespour A, Salehi R, Kasra Kermanshahi R (2011) *Lactobacillus Acidophilus-*Derived Biosurfactant Effect on *gtfB* and *gtfC* Expression Level in *Streptococcus Mutans* Biofilm Cells. Braz J Microbiol 42(1):330-9 doi:10.1590/s1517-83822011000100042

Wen ZT, Yates D, Ahn SJ, Burne RA (2010) Biofilm formation and virulence expression by *Streptococcus mutans* are altered when grown in dual-species model. BMC Microbiol 10:111 doi:10.1186/1471-2180-10-111
